# Supplementary material for: Privacy guarantees for personal mobility data in humanitarian response
Source: Sci Rep. 2024 Nov 19;14:28565. doi: 10.1038/s41598-024-79561-2 (PMC11574092; doi:10.1038/s41598-024-79561-2)
Supplement: Supplementary file 1 — Supplementary Information. [file 41598_2024_79561_MOESM1_ESM.pdf]

Supplemental Information

Privacy Guarantees for Personal Mobility Data in Humanitarian Response

Nitin Kohli, Emily Aiken, and Joshua Blumenstock

Corresponding to: jblumenstock@berkeley.edu

Contents

1 Supplementary Note: Differential Privacy **1**

1.1 Mathematical Preliminaries 1

1.2 Private O-D Matrix Algorithm 2

1.3 Privacy-Accuracy Tradeoff Theorems 3

2 Supplementary Note: Membership Inference Attack **6**

3 Supplementary Figures and Tables **8**

4 References **12**

1 Supplementary Note: Differential Privacy

In this section, we provide the mathematical foundation of Private O-D Matrix Algorithm, as well as formalize and prove all the theorems referenced in the main manuscript. In order to do so, we begin by first presenting the necessary mathematical preliminaries on differential privacy in Section 1.1. We then leverage these preliminaries to describe and formalize the design of our algorithm in Section 1.2. And lastly, in Section 1.3 we provide formal proofs of of our privacy-accuracy tradeoff theorems.

1.1 Mathematical Preliminaries

Differential privacy provides a mathematical guarantee that a privacy-preserving statistic $A(d)$ that approximates a non-private statistic $M(d)$ derived from dataset d will not reveal “too much" information about any underlying observation in dataset d [2].

Definition 1. (ϵ-Differential Privacy [3]) A randomized algorithm A mapping datasets in $\mathbb{D}$ to an outcome space $\mathbb{O}$ satisfies ϵ-differential privacy if, for all datasets $d, d' \in \mathbb{D}$ that differ in one element, and for all events $O \subseteq \mathbb{O}$, $\mathbb{P}(A(d) \in O) \leq \exp(\epsilon)\mathbb{P}(A(d') \in O)$.

Thus the privacy preserving statistic $A(d)$ is the output of a randomized algorithm A whose noisy-behavior is governed by the parameter ϵ.

It is worth noting that differential privacy is not a particular technique. Rather, it is a mathematical standard that an algorithm either satisfies or not [2]. As such, differential privacy provides us with a promise about the worst-case privacy loss incurred from running a computational process. As a general matter however, it does not tell us how to implement the promise. There are many possible ways to construct an algorithm to achieve the privacy guarantee. A foundational technique for privatizing a statistic $S : \mathbb{D} \to \mathbb{R}^m$ is

to use noisy values sampled from a Laplace distribution[1] with a carefully crafted scale parameter λ (denoted as $Lap(\lambda)$) to ensure that contribution of *any* possible data point is masked.

Lemma 1 (Laplace Mechanism [3])**.** *For a statistic $S : \mathbb{D} \to \mathbb{R}^m$, let Δ_S be the supremum of $||S(d) - S(d')||_1$, where $d, d' \in \mathbb{D}$ are datasets that differ in one element. For any $\Delta_S \in (0, \infty)$, the algorithm $A(d) = S(d) + (\zeta_1, ..., \zeta_m)^T$, where each ζ_i is drawn independently from $Lap(\epsilon^{-1}\Delta_S)$, satisfies ϵ-differential privacy.*

It is also worth highlighting that the definition of differential privacy quantifies over datasets that differ in one *element*. There are two ways to consider this elemental difference in our setting, and each has a different consequence for the level of privacy afforded. When the elemental difference in mobility datasets is a trip, differential privacy affords *trip-level privacy protection* by protecting each trip with parameter ϵ. Hence, if an individual took t trips, they experience $t\epsilon$ privacy loss. This can result in *heterogeneous privacy loss* for data subjects, with individuals who travel more incurring larger privacy losses. Alternatively, when the elemental difference in datasets is an individual, differential privacy protects all the trips an individual took with parameter ϵ, yielding *homogeneous privacy loss* for all data subjects. This *individual-level privacy protection* is a stronger privacy guarantee, but requires more noise in order to hide multiple trips.

Differentially private algorithms are accompanied with a host of provable guarantees that enable rigorous reasoning about privacy losses that occur from the computation of statistics. Three important properties for this study are the *composition result*, the *post-processing result*, and the *group privacy*.

Lemma 2 (Composition [4])**.** *Given any collection of ϵ_i-differentially private algorithms $A_i : \mathbb{D} \to \mathbb{O}_i$, the algorithm $A = (A_1, ..., A_r) : \mathbb{D} \to \mathbb{O}_1 \times ... \times \mathbb{O}_r$ is ($\epsilon_1 + ... + \epsilon_r$)-differentially private.*

Lemma 3 (Group-Privacy [4])**.** *Suppose $d, d' \in \mathbb{D}$ are datasets that differ in g elements. Then any ϵ-differentially private algorithm $A : \mathbb{D} \to \mathbb{O}$ further guarantees that $\mathbb{P}(A(d) \in O) \leq \exp(g\epsilon)\mathbb{P}(A(d') \in O)$ for all events $O \subseteq \mathbb{O}$.*

Lemma 4 (Post-Processing [4])**.** *Given an ϵ-differentially private algorithm $A : \mathbb{D} \to \mathbb{O}$ and any (potentially randomized) function $f : \mathbb{O} \to \mathbb{O}'$, $f \circ A$ is still ϵ-differentially private.*

Practically speaking, if humanitarian response requires multiple computations to be run on the data, then the composition result allows us to keep track of the total privacy loss accumulated by adding up the ϵ's used. Additionally, if the output of a differentially private analysis subsequently needs to be analyzed or manipulated in any way without the original data, then the post-processing result ensures no additionally privacy loss has occurred. And lastly, the group privacy result informs us that: (1) when trip-level privacy is used, the total privacy incurred scales linearly with the number of trips g that an individual took; and (2) when individual-level privacy is used, the total privacy incurred by a group of g individuals scales linearly as well.

1.2 Private O-D Matrix Algorithm

For clarity of exposition, we redisplay Algorithm 1 that was presented in the main manuscript. Using the mathematical tools from Section 1.1, we deduce that our algorithm satisfies ϵ-differential privacy.

Theorem 1. *For any value $\tau \in \mathbb{Z}_{\geq 0}$ that is selected independent of data, the Private O-D Matrix algorithm satisfies (1) individual-level ϵ-differential privacy for any $T \in \mathbb{Z}_{\geq 1}$ that is also determined independently of the underlying data, and (2) trip-level ϵ-differential privacy when $T = 1$.*

Proof. It is sufficient to privatize the non-diagonal entries of $M(d)$, as these are the only entries that are responsive to d. When individual-level privacy is required, the inclusion or deletion of any record in d can change the sum of the non-diagonal entries of $M(d)$ by $\pm T$. And when trip-level privacy is required, any record in d can change the sum of the non-diagonal entries of $M(d)$ by ± 1 (which equals $\pm T$ as per the algorithm). Hence, $\Delta_M = T$. Adding $k^2 - k$ independently sampled $Lap(\epsilon^{-1}T)$ values to the non-diagonal entries of $M(d)$ is equivalent to reshaping the non-diagonal entries of $M(d)$ as a vector $S(d) \in \mathbb{R}^{k^2-k}$ and then utilizing the Laplace mechanism, yielding ϵ-differential privacy. After this, there is no additional privacy loss by the post-processing result, as τ was set agnostic of d. □

[1]The Laplace distribution with scale parameter $\lambda \in (0, \infty)$ corresponds to a real-valued random variable with probability density function $f(z) = \frac{1}{2\lambda}\exp(-|z|/\lambda)$ [3]. This distribution has mean 0 and a standard deviation of $\sqrt{2}\lambda$.

Algorithm 1: Private O-D Matrix

Input:

- Dataset d
- Privacy parameter $\epsilon \geq 0$
- Trip threshold $T \in \mathbb{Z}_{\geq 1}$: For individual-level differential privacy, T corresponds to the maximum number of trips an individual can contribute the the non-private O-D matrix; the dataset d should be preprocessed to ensure every individual contributes at most T trips. For trip-level differential privacy, set $T = 1$; no additional preprocessing of d is required.
- Suppression threshold $\tau \in \mathbb{Z}_{\geq 0}$

Output: ϵ-differentially private O-D matrix $\hat{M}(d)$

1 (*Non-private computation*) For a geographic region with k zones, compute the $k \times k$ matrix $M(d)$, where $M(d)_{a,b}$ equals the number of trips from zone a to zone b when $a \neq b$, and 0 when $a = b$.

2 (*Incorporate privacy*) Add independent Laplace noise with scale $\lambda = \epsilon^{-1}T$ to each non-diagonal entry of $M(d)$. Call this resulting matrix $M'(d)$.

3 (*Post-processing*) Next,
(I) Round each of the resulting entries in $M'(d)$ to the nearest integer. In the event where there is no unique nearest integer, we adopt the convention of rounding up to the nearest integer.
(II) Then, for all resulting values that are below a threshold of τ, map these values to 0.
Call this resulting matrix $\hat{M}(d)$

4 **Return** $\hat{M}(d)$

1.3 Privacy-Accuracy Tradeoff Theorems

Our first two privacy-accuracy tradeoff theorems demonstrate that our algorithm preserves suppression and non-suppression of the non-private entries with high probability.

To do so, we consider a dataset d that produces a non-private matrix $M(d)$ and a private matrix $\hat{M}(d)$ using some threshold τ and some privacy parameter ϵ. Unless $\tau = 0$ we cannot directly compare these two outputs, as the private matrix will not contain any counts between 1 and $\tau - 1$ due to the suppression. For an apples-to-apples comparison, let $\bar{M}(d)$ be the result of mapping all entries in $M(d)$ that are below τ to 0.

Theorem 2. *For any $T \in \mathbb{Z}_{\geq 1}$, $\tau \in \mathbb{Z}_{\geq 0}$, and for any suppressed non-diagonal entry (a, b) in $\bar{M}(d)$, $\hat{M}(d)_{a,b}$ is also suppressed with probability* $1 - 0.5\exp(-\epsilon T^{-1}(\tau - 0.5 - M(d)_{a,b}))$.

Proof. $\bar{M}(d)_{a,b}$ is suppressed $\iff$ $M(d)_{a,b} < \tau$. For $\eta \sim \mathrm{Lap}(\epsilon^{-1}T)$, we have $\mathbb{P}(\hat{M}(d)_{a,b} = 0) = \mathbb{P}(\mathrm{round}(M(d)_{a,b} + \eta) < \tau) = \mathbb{P}(\eta < \tau - 0.5 - M(d)_{a,b}) = 1 - 0.5\exp(-\epsilon T^{-1}(\tau - 0.5 - M(d)_{a,b}))$. □

Theorem 3. *For any $T \in \mathbb{Z}_{\geq 1}$, $\tau \in \mathbb{Z}_{\geq 0}$, and for any non-suppressed non-diagonal entry (a, b) of $\bar{M}(d)$, $\hat{M}(d)_{a,b}$ is also not suppressed with probability* $1 - 0.5\exp(\epsilon T^{-1}(\tau + 0.5 - M(d)_{a,b}))$.

Proof. $\bar{M}(d)_{a,b}$ is not suppressed $\iff$ $M(d)_{a,b} \geq \tau$. For $\eta \sim \mathrm{Lap}(\epsilon^{-1}T)$, $\mathbb{P}(\hat{M}(d)_{a,b} \neq 0) = \mathbb{P}(\mathrm{round}(M(d)_{a,b} + \eta) \geq \tau) = \mathbb{P}(\eta \geq \tau + 0.5 - M(d)_{a,b}) = 1 - 0.5\exp(\epsilon T^{-1}(\tau + 0.5 - M(d)_{a,b}))$. □

Our third privacy-accuracy tradeoff theorem quantifies the probability that the error between a non-private matrix entry and private matrix entry exceeds α, in cases where the (a, b) entry is non-suppressed in both the non-private and private matrices.

Theorem 4. *Suppose (a, b) is a non-suppressed non-diagonal entry of both $M(d)$ and $\hat{M}(d)$. Then for any $\alpha \in \mathbb{Z}_{\geq 0}$, the chance that $|\hat{M}(d)_{a,b} - M(d)_{a,b}| > \alpha$ is* $\exp(-\epsilon T^{-1}(\alpha + 0.5))$.

Proof. For $\eta \sim \mathrm{Lap}(\epsilon^{-1}T)$ and $\alpha \in \mathbb{Z}_{\geq 0}$, $\mathbb{P}(|\hat{M}(d)_{a,b} - M(d)_{a,b})| > \alpha) = \mathbb{P}(|\mathrm{round}(\eta)| > \alpha) = 2\mathbb{P}(\eta \geq \alpha + 0.5) = \exp(-\epsilon T^{-1}(\alpha + 0.5))$. □

The previous three results can be viewed as demonstrating "static" accuracy guarantees. However, mobility matrices can be used to inform policy decisions based on differences in population flow at two points in time. Our privatization method can still allow for strong accuracy guarantees, even when looking at such differences. For any non-diagonal entry (a, b), let $M_{t_1}(d)_{a,b}$ and $M_{t_2}(d)_{a,b}$ be represent the non-private counts of trips from a to b at time periods t_1 and t_2 respectively. The following privacy-accuracy tradeoff theorem allows us to quantify the chance that the observed difference in the entry (a, b) in private matrices and the actual difference the (a, b) entry of non-private matrices is larger than α.

Theorem 5. *Suppose the counts in $M_{t_1}(d)_{a,b}, M_{t_2}(d)_{a,b}, \hat{M}_{t_1}(d)_{a,b}$, and $\hat{M}_{t_2}(d)_{a,b}$ are all non-suppressed. Then for any $\alpha \in \mathbb{Z}_{\geq 0}$ and $T \in \mathbb{Z}_{\geq 1}$, the chance that $|(\hat{M}_{t_2}(d)_{a,b} - \hat{M}_{t_1}(d)_{a,b}) - (M_{t_2}(d)_{a,b} - M_{t_1}(d)_{a,b})| > \alpha$ is given by*

$$\exp(-\epsilon T^{-1}(\alpha+1))\frac{\epsilon T^{-1}(\alpha+1)+2}{2}$$

To streamline the proof of the Theorem 5, we first provide a helpful technical lemma.

Lemma 5. *For any $\alpha \in \mathbb{R}$ and independent $\eta_1, \eta_2 \sim Lap(\epsilon^{-1}T)$,*

$$\mathbb{P}(\eta_2 - \eta_1 \geq \alpha) = \exp(-\text{sgn}(\alpha)\epsilon\alpha T^{-1})\frac{\epsilon\alpha T^{-1} + 2(\text{sgn}(\alpha) + \mathbb{I}(\alpha = 0))}{4}$$

Proof. Each $\eta_i \sim Lap(\epsilon^{-1}T)$, so they can be written as the difference of two independent Exponential random variables with rate parameter ϵT^{-1}. So $\eta_2 - \eta_1$ can be written as the difference of independent Gamma random variables X' and Y', each with shape parameter 2 and rate parameter ϵT^{-1}. To simplify calculations, we rewrite $X' = (\epsilon^{-1}T)X$ and $Y' = (\epsilon^{-1}T)Y$, where X and Y independent Gammas, each with shape 2 and rate 1. Hence, $\eta_2 - \eta_1 = (\epsilon^{-1}T)(X - Y)$. The pdf for a Gamma distribution with shape 2 and rate 1 is given by $f(t) = t\exp(-t)$. So, when $z < 0$,

$$f_{X-Y}(z) = \int_0^\infty f_X(x)f_Y(x-z)dx = \frac{1}{4}\exp(z)(1-z)$$

Since $X - Y$ is symmetric about 0, when $z \geq 0$ we deduce from the above integration that $f_{X-Y}(z) = \frac{1}{4}\exp(-z)(1+z)$. Hence, $f_{X-Y}(z) = \frac{1}{4}\exp(-|z|)(1+|z|)$ for all $z \in \mathbb{R}$, so then

$$\mathbb{P}(\theta_2 - \theta_1 \geq \alpha) = \mathbb{P}(X - Y \geq \epsilon\alpha T^{-1}) = \int_{\epsilon\alpha T^{-1}}^\infty \frac{1}{4}\exp(-|z|)(1+|z|)dz$$

When $\alpha \geq 0$,

$$\mathbb{P}(\theta_2 - \theta_1 \geq \alpha) = \int_{\epsilon\alpha T^{-1}}^\infty \frac{1}{4}\exp(-z)(1+z)dz = \exp(-\epsilon\alpha T^{-1})\frac{\epsilon\alpha T^{-1}+2}{4}$$

And when $\alpha < 0$,

$$\mathbb{P}(\theta_2 - \theta_1 \geq \alpha) = \int_{\epsilon\alpha T^{-1}}^0 \frac{1}{4}\exp(z)(1-z)dz + \frac{1}{2} = \exp(\epsilon\alpha T^{-1})\frac{\epsilon\alpha T^{-1}-2}{4}$$

Consolidating these cases delivers the advertised claim. □

Proof of Theorem 5

Proof. Let $D_{1,2} := (\hat{M}_{t_2}(d)_{a,b} - \hat{M}_{t_1}(d)_{a,b}) - (M_{t_2}(d)_{a,b} - M_{t_1}(d)_{a,b})$. By construction,

$$\hat{M}_{t_j}(d)_{a,b} = \text{round}(M_{t_j}(d)_{a,b} + \eta_j)$$

for independently drawn $\eta_j \sim Lap(\epsilon^{-1}T)$ for $j \in \{1, 2\}$. Then $D_{1,2} = \text{round}(\eta_2) - \text{round}(\eta_1)$. As η_1 and η_2 are identically distributed, so too are round(η_1) and round(η_2). So then (round(η_2) − round(η_1)) and (round(η_1) − round(η_2)) are also identically distributed. Thus,

$$\mathbb{P}(|D_{1,2}| > \alpha) = \mathbb{P}(|\text{round}(\eta_2) - \text{round}(\eta_1)| > \alpha) = 2\mathbb{P}(\text{round}(\eta_2) - \text{round}(\eta_1) > \alpha) = 2\mathbb{P}(\eta_2 - \eta_1 \geq \alpha + 1)$$

Replacing α with $(\alpha + 1)$ from Lemma 5 completes the proof. □

These privacy-accuracy tradeoff theorems described above can be leveraged to *proactively* enable policymakers to set ϵ based on context-specific goals. In particular, we derive *exact formulas* to set ϵ in Theorems 6 and 7. To elucidate discussion, suppose policymakers can tolerate some maximal error α with confidence $100(1-\beta)\%$ and still provide effective interventions. Then we can set $\beta \geq \exp(-\epsilon T^{-1}(\alpha + 0.5))$. Solving for ϵ yields $\epsilon \geq -T(\alpha + 0.5)^{-1}\ln(\beta)$. This yields the following result.

Theorem 6. *Any $\beta \in [0, 1)$ upper-bounds the result from Theorem 4 $\iff \epsilon \geq -T(\alpha + 0.5)^{-1}\ln(\beta)$.*

Alternatively, if policymakers are instead interested in ensuring that trends across different O-D matrices are α-inaccurate with $100(1-\beta)\%$ confidence, then we can use Theorem 5 to derive feasible values of ϵ to meet this goal using the Lambert-W function[2] [1].

Theorem 7. *For $\beta \in [0, 0.5\exp(-1)]$, β upper-bounds the result from Theorem 5 $\iff \epsilon \geq T(\alpha+1)^{-1}(-2 - W_{-1}(-2\beta\exp(-2)))$ where W_{-1} is the lower branch of the Lambert-W function.*

Proof. Let $A = -\epsilon T^{-1}(\alpha + 1) - 2$. Then,

$$\beta \geq \exp(-\epsilon T^{-1}(\alpha + 1))\frac{\epsilon T^{-1}(\alpha + 1) + 2}{2} \iff -2\beta\exp(-2) \leq A\exp(A)$$

Since $\beta \leq 0.5\exp(-1)$, we have $-2\beta\exp(-2)) \geq -\exp(-1)$, so $-2\beta\exp(-2))$ is within the domain of the Lambert-W function. Since $-2\beta\exp(-2)) < 0$ there are two potential solutions for A over the reals: one involving the principal branch W_0 and a second involving the lower branch W_{-1}.

For the first case, we will show that there is no feasible positive ϵ that works. Since W_0 is an increasing function, $-2\beta\exp(-2) \leq A\exp(A) \iff W_0(-2\beta\exp(-2)) \leq A \iff \epsilon \leq T(\alpha + 1)^{-1}(-2 - W_0(-2\beta\exp(-2)))$. But this bound is always negative, as $-2\beta\exp(-2) < 0$ implies $W_0(-2\beta\exp(-2)) < 0$. So there is no feasible positive ϵ from the use of the principal branch.

For the second case, we will derive the inequality stated in the theorem. As W_{-1} is a decreasing function, $-2\beta\exp(-2) \leq A\exp(A) \iff W_{-1}(-2\beta\exp(-2)) \geq A \iff \epsilon \geq T(\alpha + 1)^{-1}(-2 - W_{-1}(-2\beta\exp(-2)))$. Unlike the prior case, this bound is indeed a positive quantity. By definition of the Lambert-W function, $W_{-1}(-2\exp(-2)) = -2$. Since $\beta \leq 0.5\exp(-1) < 1$, we have $-2\exp(-2) < -2\beta\exp(-2)$. Since W_{-1} is decreasing, we deduce that $-2 = W_{-1}(-2\exp(-2)) \geq W_{-1}(-2\beta\exp(-2))$ which implies that the bound is positive. □

[2] The Lambert-W function is a countable family of functions $\{W_k : \mathbb{C} \to \mathbb{C} \mid k \in \mathbb{Z}\}$ that satisfies the product-exponential equation $x = W_k(x)\exp(W_k(x))$. That is, for $x, y \in \mathbb{C}$, $x = y\exp(y) \iff y = W_k(x)$ for some $k \in \mathbb{Z}$. In the language of complex analysis, each W_k is a branch of the Lambert-W function. When x is restricted to the real line instead of the complex plane, the domain of the Lambert-W function is $x \geq -\exp(-1)$. When $x < 0$ as well, it is sufficient to consider the principal branch W_0 and the lower branch W_{-1} to solve for $y \in \mathbb{R}$. The principal branch W_0 corresponds to all x such that $W_0(x) \geq -1$, while the lower branch W_{-1} corresponds to all x such that $W_{-1}(x) \leq -1$. See [1] for a further mathematical details.

2 Supplementary Note: Membership Inference Attack

We implement a membership inference attack (MIA) to assess (1) the extent to which aggregated mobility matrices leak individual information that can be exposed by adversaries, and (2) the extent to which differentially private matrices protect this information. We implement the membership inference attack from Pyrgelis et al. (2017) [5], which uses machine learning tools to attempt to *predict* whether an individual's information is included in an aggregate mobility trace at time t based on the mobility aggregate itself. The machine learning model is trained on previous mobility traces at time $t^* < t$ — both traces that do and do not include data from the target individual — where the adversary has knowledge of the "label" for the ML model (whether or not the target subscriber's information is included in the aggregate).

Our version of the membership inference attack corresponds to attack version 2A from Pyrgelis et al. (2017), where the adversary has knowledge of historic participation in past groups of a set of individuals, including the target individual. We believe this version of the attack most closely corresponds to our setting: in our setting, the adversary may have knowledge about a target subscriber's previous movements (between the same set of regions) if they have access to historical mobile phone metadata at the individual level.

We implement the attack as follows, using our Afghanistan 2020 dataset, which includes 305 days with mobility data. Our other two datasets — which are each one week long — are prohibitively short to provide sufficient observations for training a machine learning model for the attack. We focus on the Afghanistan data at the province level, to keep the experiments at a reasonable dimensionality (at the province level there are 34 x 34 = 1,156 OD matrix counts to use as input features to the machine learning model; at the district level there would be 421 x 421 = 177,241 features to use).

1. Divide the time series (305 days) into disjoint training sets (the first half of the time period, 152 days) and test sets (the second half of the time period, 153 days).

2. Sample 100 subscribers at random from the set of subscribers with trips in the aggregate mobility matrices, restricting to subscribers that make at least 10 trips during the training period and 10 trips during the test period (to ensure that there are at least 10 positive-instance trips to train the ML model on, and at least 10 positive-instance trips to use for evaluation). These are the subscribers we will run the MIA against. The choice of 100 subscribers is similar to the 150 total individuals that Pyrgelis et al. (2017) uses for the attack.

3. For each of the 100 sampled subscribers, identify whether they are "in" or "out" of the OD matrix each day of the training and test sets (i.e. whether they make at least one trip that appears in an OD matrix count on the day in question). Thus, for each subscriber, there are 152 observations for which they are either "in" or "out" of the OD matrix in the training set and 153 observations for which they are either "in" or "out" of the OD matrix in the test set. This indicator for whether a subscriber is "in" or "out" of the matrix is the predictive target of the ML model (see #5 below).

4. Following Pyrgelis et al. (2017) we create a balanced sample in the training and test sets. To produce this balanced sample we downsample negative observations per subscriber: we keep all of the subscriber's positive observations (instances where they are "in" a specific OD matrix count) and sample the same number of negative observations. Subscribers in our sample make trips on between 20 and 150 days over the duration of the entire period (train and test set together), with a mean of 47 and a median of 32.

5. The input features for the machine learning model are all 34 provinces $\times$ 34 provinces = 1,156 OD matrix counts for the day of the observation (either nonprivate or differentially private). For each subscriber separately, we train a machine learning classifier on the training set to predict whether the subscriber is "in" the OD matrix in question from the input features. We then produce predictions for the test set, and calculate the area under the curve (AUC) score on the test set. For machine learning models, we experiment with a logistic regression (with L1 penalty chosen via three fold cross validation) and a random forest (with an ensemble size of 100 and maximum depth chosen via three fold cross validation).

6. We test the MIA in three settings: (1) using nonprivatized mobility matrices as inputs, (2) using privatized mobility matrices as inputs in both the training and test sets, and (3) using nonprivated

mobility matrices in training and privatized mobility matrices in testing. The third option is inspired by Pyrgelis et al.'s (2017) finding that while an adversary that trains on raw data is likely to be inhibited by privatized matrices in the test set, an adversary that mimics the perturbation of mobility matrices in the test set by privatizing the training set will be more successful.

7. We also test MIA with additional time-related information (in addition to OD matrix counts) as inputs to the model. We consider the inclusion of day-of-week fixed effects to allow the adversary to take advantage of weekly cyclical mobility patterns in inference.

Table 3 provides the results of our experiments with the membership inference attack. We find that the ML-based adversarial approach does have some ability to predict whether individual subscribers are included in an OD matrix from the nonprivatized aggregated counts (average AUC = 0.61-0.62 for a logistic regression 0.63-0.64 for a random forest). Adding time fixed effects does not improve the accuracy of the adversary. In comparison to the two mobility datasets from taxi drivers and subway systems studied in Pyrgelis et al. (AUC = 0.81-0.99), the performance of the adversary in our setting is much lower. This may be because the number of individuals underlying our dataset is much larger: our 305-day Afghanistan dataset includes transactions from around 7 million subscribers, whereas the two datasets analyzed in Pyrgelis et al. (2017) include data from 536 and 10,000 individuals.

In our setting differential privacy provides a small amount of additional privacy protection in the setting where the model is trained on raw (nonprivatized OD matrices). In particular, the random forest in this setting achieves an AUC of 0.623-0.624 in comparison to an AUC of 0.631 for a model evaluated on nonprivatized data. Consistent with the results in Pyrgelis et al. (2017), we find that these privacy gains are attenuated when the model is trained on privatized matrices and can learn to mimic the pattern of noise injected (AUC = 0.633). Across the board, we see no difference between attacks using privated and nonprivatized matrices when the logistic regression is used for the attack (although the attacker's accuracy, even with nonprivatized matrices, is lower for the logistic regression than for the random forest).

3 Supplementary Figures and Tables

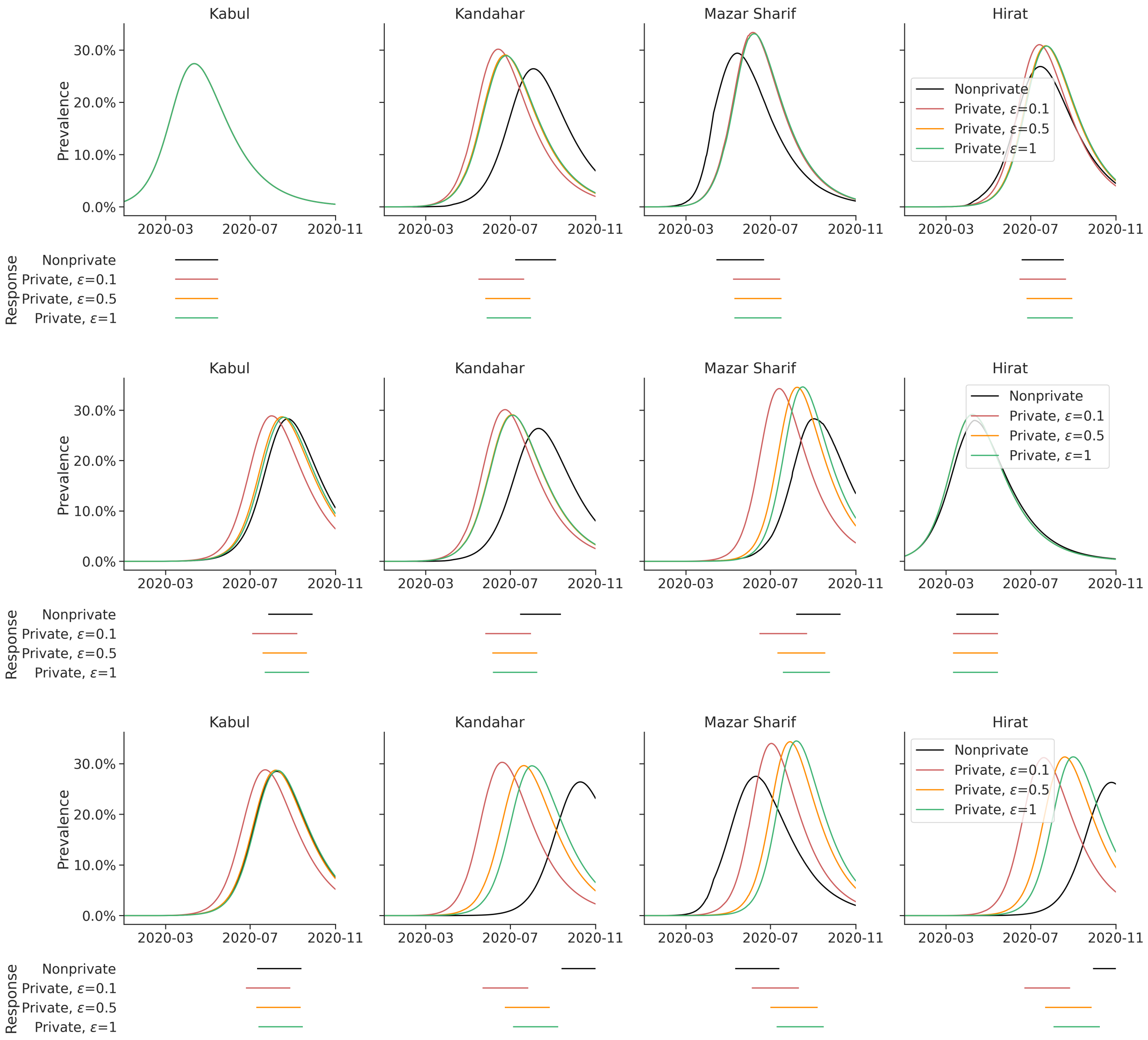

Figure S1: Epidemic curves based on mobility-based SIR models and O-D matrices derived from call detail records. Top: Pandemic initiating in Kabul. Middle: Pandemic initiating in Hirat. Bottom: Pandemic initiating randomly.

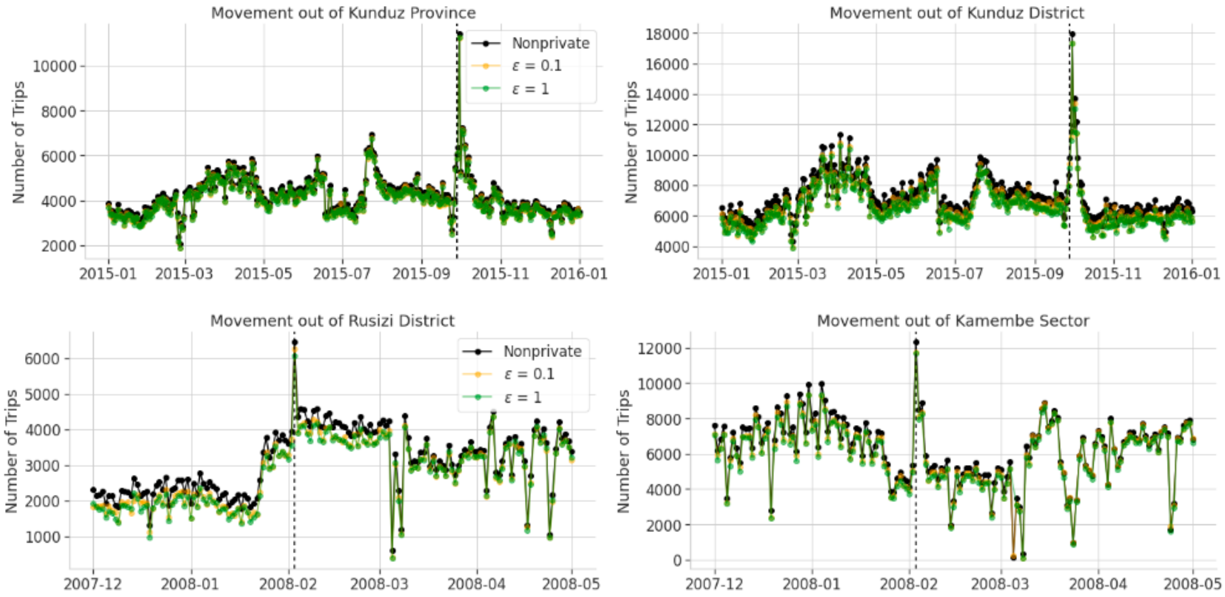

Figure S2: Total out-migration from areas used in simulations of humanitarian response to natural disasters and violent events, calculated from call detail records. Top: Out-migration during the Battle of Kunduz in Afghanistan. Bottom: Out-migration following the Lake Kivu Earthquake in Rwanda.

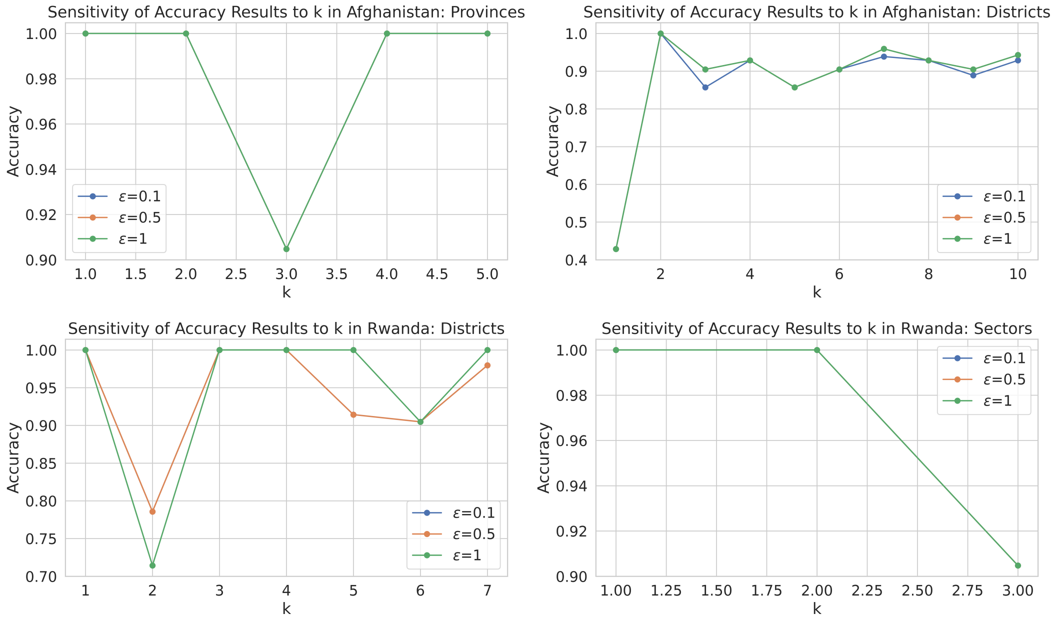

Figure S3: Sensitivity of results on accuracy for identifying top-k regions of out migration in simulations of natural disasters and violent events to value of k. Left: admin-2 level. Right: admin-3 level. Lines are not present where there are fewer than k regions of out-migration after suppression of small counts.

	Features: OD counts only		Features: OD counts and weekday dummies	
	Logistic regression	***Random forest***	***Logistic regression***	***Random forest***
Panel A: Train on non-private data				
**Non-private**	0.619	0.643	0.619	0.631
ϵ = **0.1**	0.619	0.628	0.619	0.623
ϵ = **0.5**	0.620	0.626	0.620	0.624
ϵ = **1**	0.620	0.626	0.620	0.624
Panel B: Train on private data				
**Non-private**	0.619	0.643	0.619	0.631
ϵ = **0.1**	0.614	0.636	0.614	0.633
ϵ = **0.5**	0.614	0.634	0.614	0.633
ϵ = **1**	0.614	0.634	0.614	0.633

Table S1: Results of membership inference attack on raw OD matrices vs. privatized matrices (with three different values of ϵ using trip-level privacy). The attack is run on our 305-day Afghanistan dataset. The first panel trains the attack model on raw OD matrices; the second panel trains the attack model on privatized matrices. Each table cell represents the average AUC score for the attack in the test set on 100 sampled subscribers.

	Rwanda 2008		Afghanistan 2015		Afghanistan 2020	
	**Districts**	**Sectors**	**Provinces**	**Districts**	**Provinces**	**Districts**
**Non-private**	49.3%	78.1%	75.9%	96.7%	73.6%	97.8%
ϵ = **0.1**	47.6%	88.3%	65.9%	88.2%	64.1%	88.2%
ϵ = **0.5**	49.2%	90.6%	75.7%	97.1%	73.5%	97.7%
ϵ = **1.0**	49.4%	78.0%	76.0%	96.5%	73.6%	97.5%

Table S2: Share of OD matrix counts that have fewer than 15 trips in each dataset. For privatized data, these regions are suppressed in our empirical simulations — that is, their OD matrix counts are set to 0. The large number of OD matrix counts with fewer than 15 trips across datasets reflects the sparsity of the OD matrices: many region-to-region pairs have little direct travel, particularly those that are far apart from one another.

	Admin-2 level				Admin-3 level			
	Non-Private	ϵ=0.1	ϵ=0.5	ϵ=1	*Non-private*	ϵ=0.1	ϵ=0.5	ϵ=1
Panel A: Battle of Kunduz in Afghanistan, T set at 99th percentile of daily trips								
Total out-migration	49,994	42,301	40,982	40,920	87,007	83,471	74,755	74,507
% error in total out-migration	0.00%	15.39%	18.03%	18.15%	0.00%	4.06%	14.08%	14.37%
Acc. of top-k regions (k = 3)	100.00%	85.71%	85.71%	85.71%	100.00%	85.71%	85.71%	85.71%
Panel B: Battle of Kunduz in Afghanistan, T set at 95th percentile of daily trips								
Total out-migration	49,994	41,492	40,173	40,111	87,007	79,995	71,298	71,035
% error in total out-migration	0.00%	17.01%	19.64%	19.77%	0.00%	8.06%	18.05%	18.36%
Acc. of top-k regions (k = 3)	100.00%	85.71%	85.71%	85.71%	100.00%	85.71%	85.71%	85.71%
Panel C: Battle of Kunduz in Afghanistan, T set at 90th percentile of daily trips								
Total out-migration	49,994	40,389	39,070	39,008	87,007	75,157	66,374	66,144
% error in total out-migration	0.00%	19.21%	21.85%	21.97%	0.00%	13.62%	23.71%	23.98%
Acc. of top-k regions (k = 3)	100.00%	85.71%	85.71%	85.71%	100.00%	85.71%	85.71%	85.71%
Panel D: Lake Kivu Earthquake in Rwanda, T set at 99th percentile of daily trips								
Total out-migration	32,627	30,710	30,752	30,740	51,102	57,657	47,504	47,259
% error in total out-migration	0.00%	5.00%	4.87%	4.91%	0.00%	12.83%	9.00%	7.52%
Acc. of top-k regions (k = 3)	100.00%	100.00%	100.00%	100.00%	100.00%	80.95%	85.71%	85.71%
Panel E: Lake Kivu Earthquake in Rwanda, T set at 95th percentile of daily trips								
Total out-migration	32,627	29,823	29,880	29,883	51,102	56,365	46,231	45,992
% error in total out-migration	0.00%	8.59%	8.42%	8.41%	0.00%	10.30%	9.53%	10.00%
Acc. of top-k regions (k = 3)	100.00%	100.00%	100.00%	100.00%	100.00%	85.71%	85.71%	85.71%
Panel E: Lake Kivu Earthquake in Rwanda, T set at 90th percentile of daily trips								
Total out-migration	32,627	28,819	28,866	28,868	51,102	55,273	45,169	44,935
% error in total out-migration	0.00%	11.67%	11.53%	11.52%	0.00%	8.16%	11.61%	12.07%
Acc. of top-k regions (k = 3)	100.00%	100.00%	100.00%	100.00%	100.00%	85.71%	85.71%	85.71%

Table S3: Replication of Table 3 with different values of T. Recall that T represents a cut-off where, for any subscriber making more than T trips on a given day, only T of those trips are sampled for inclusion in the calculation of the OD matrix. We test three values of T for both the Battle of Kunduz in Afghanistan and the Lake Kivu earthquake in Rwanda. We take the 99th, 95th, and 90th percentiles of daily trips that subscribers make in each setting. For Afghanistan, this corresponds to T = 6, 4, and 3 at the admin-2 level and T = 10, 6, and 4 at the admin-3 level. For Rwanda, these percentiles correspond to T = 14, 8, and 6 at the admin-2 level, and T = 20, 11, and 9 at the admin-3 level.

4 References

[1] Ioannis Chatzigeorgiou. Bounds on the lambert function and their application to the outage analysis of user cooperation. *IEEE Communications Letters*, 17(8):1505–1508, 2013.

[2] Cynthia Dwork, Nitin Kohli, and Deirdre Mulligan. Differential privacy in practice: Expose your epsilons! *Journal of Privacy and Confidentiality*, 9(2), 2019.

[3] Cynthia Dwork, Frank McSherry, Kobbi Nissim, and Adam Smith. Calibrating noise to sensitivity in private data analysis. In *Theory of cryptography conference*, pages 265–284. Springer, 2006.

[4] Cynthia Dwork and Aaron Roth. The algorithmic foundations of differential privacy. *Foundations and Trends in Theoretical Computer Science*, 9(3–4):211–407, 2014.

[5] Apostolos Pyrgelis, Carmela Troncoso, and Emiliano De Cristofaro. Knock knock, who's there? membership inference on aggregate location data. *arXiv preprint arXiv:1708.06145*, 2017.
